# Supplementary material for: Controlling photophysical properties of ultrasmall conjugated polymer nanoparticles through polymer chain packing
Source: Nat Commun. 2017 May 16;8:15256. doi: 10.1038/ncomms15256 (PMC5440812; doi:10.1038/ncomms15256)
Supplement: Supplementary Information — Supplementary Figures, Supplementary Tables, Supplementary Note and Supplementary References. [file ncomms15256-s1.pdf]

**Supplementary Note 1: Estimation of the number of the spectroscopic units inside the single Pdots**

The number of the CP chains inside each PD1-L and PD2-L particle was estimated to be 28 and 444 chains/particle ,respectively, by comparison of the molar extinction coefficients ($\varepsilon$) of the CP molecules in the solution ($\varepsilon_{PCzBT}$ = 1.15 × $10^5$ and $\varepsilon_{PCzDTBT}$ = 2.7 × $10^4$) and those of the Pdots ($\varepsilon_{PD1\text{-}L}$ = 2.8 × $10^6$ and $\varepsilon_{PD2\text{-}L}$ = 1.2 × $10^7$). Each PCzBT and PCzDTBT chain respectively contains on average 7.9 and 3.3 monomers ($M_n^{PCzBT}$ = 5,400, $MW_{CzBT}$ = 684, $M_n^{PCzDTBT}$ = 2,800, $MW_{CzDTBT}$ = 848). Thus, the number of the monomers inside each PD1-L and PD2-L was estimated to be 192 and 1467 monomers/particle, respectively. Since the dimer is the spectroscopic unit of PCzBT and PCzDTBT, the number of the spectroscopic units inside each PD1-L and PD2-L particle was estimated to be 96 and 733 spectroscopic units/particle, respectively.

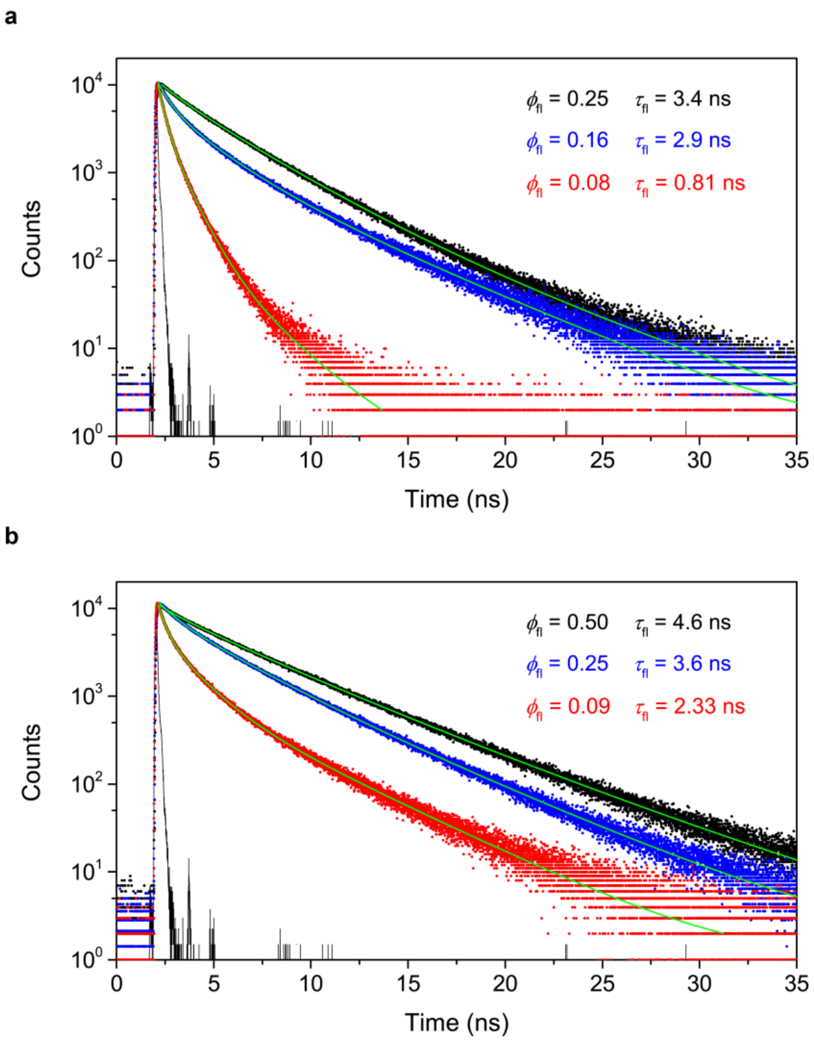


**Supplementary Figure 1. Fluorescence lifetimes of the fabricated Pdots.** (a) Bulk-phase fluorescence decay curves of PCzBT in THF (black dots), PD1-L in water (blue dots), and PD1-H in water (red dots). The green lines show fitting of the data to multi-exponential decaying functions. The grey line shows the instrument response function (IRF). (b) Bulk-phase fluorescence decay curves of PCzDTBT in THF (black dots), PD2-L in water (blue dots), and PD2-H in water (red dots). The green lines show fitting of the data to multi-exponential decaying functions. $\phi_{fl}$ and $\tau_{fl}$ are fluorescence quantum yield and mean fluorescence lifetime.

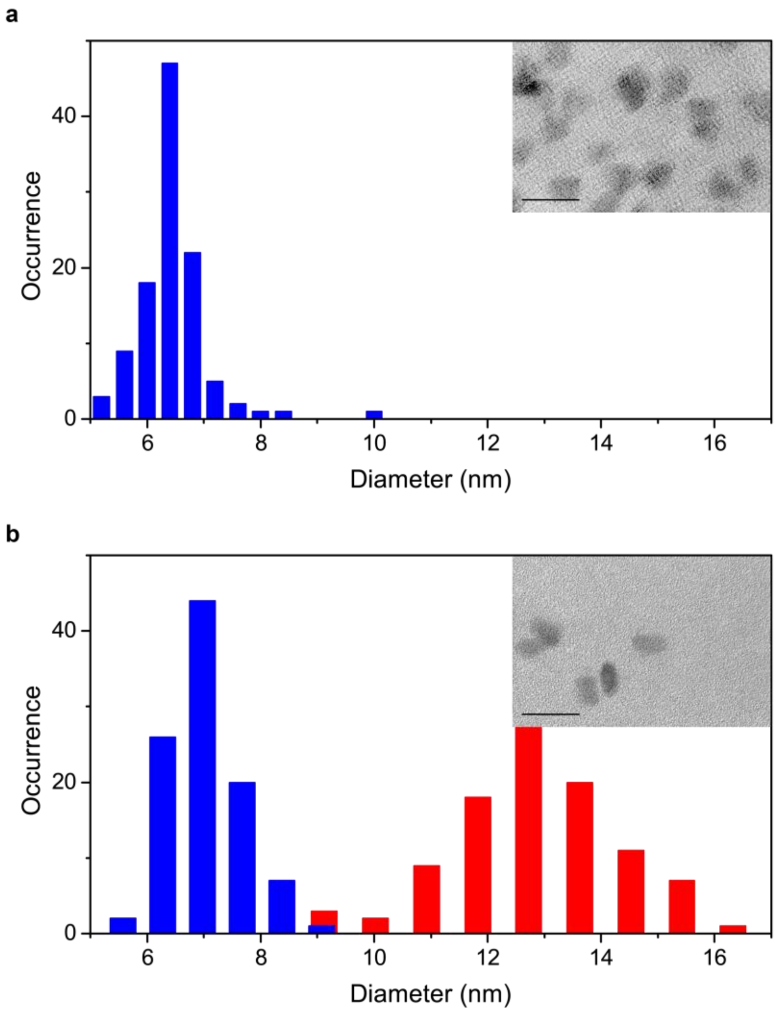


**Supplementary Figure 2. Size distributions of semiconductor quantum dots (QDs).** (a) Frequency histogram of the diameters of QD605. The inset shows a transmission electron microscopy (TEM) image of QD605. (b) Frequency histogram of the shorter (blue) and longer (red) diameters of QD655. The inset shows a TEM image of QD655. Scale bars = 20 nm.

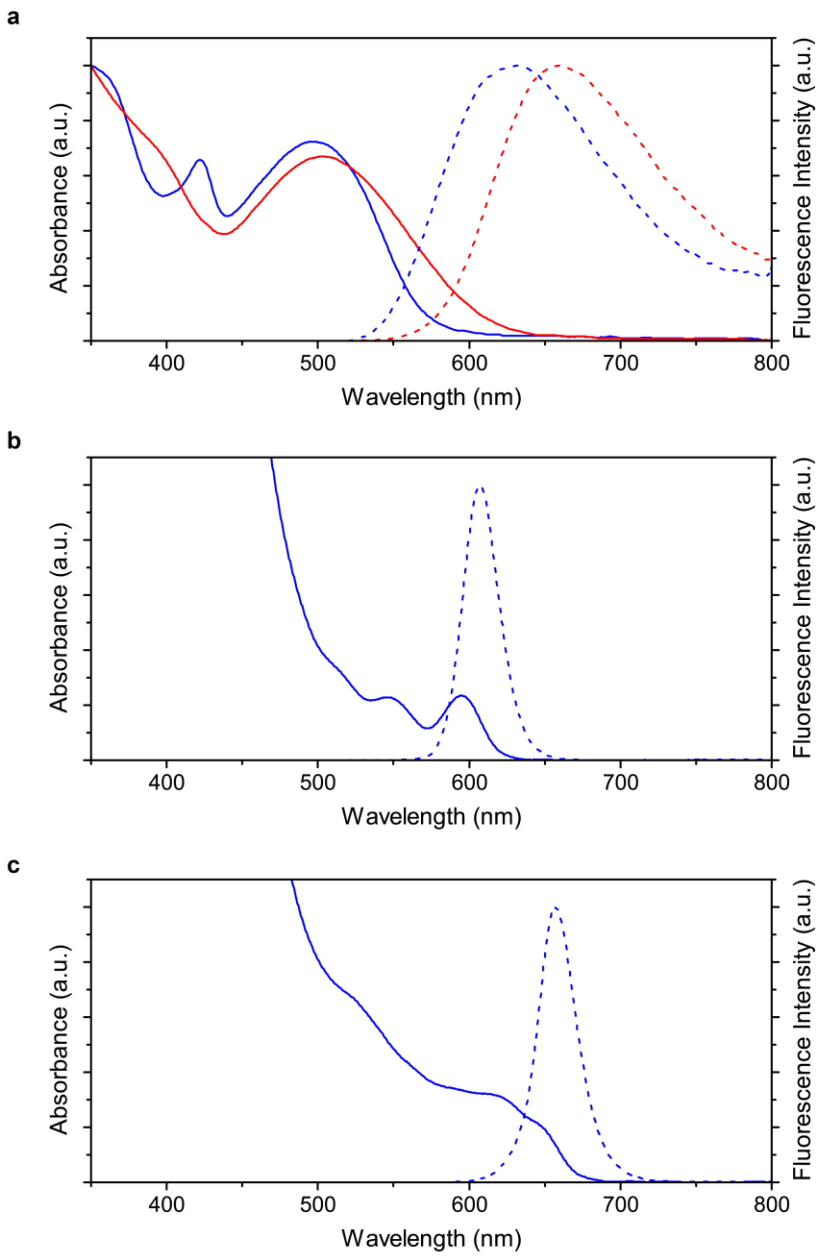


**Supplementary Figure 3. Steady-state spectra of the fabricated Pdots and QDs.** (a) Absorption (solid lines) and fluorescence (dashed lines) spectra of the Pdots fabricated at 277 K using PCzBT (PD1-L, blue lines) and PCzDTBT (PD2-L, red lines). (b) Absorption (solid line) and photoluminescence (dashed line) spectra of QD605. (c) Absorption (solid line) and photoluminescence (dashed line) spectra of QD655.

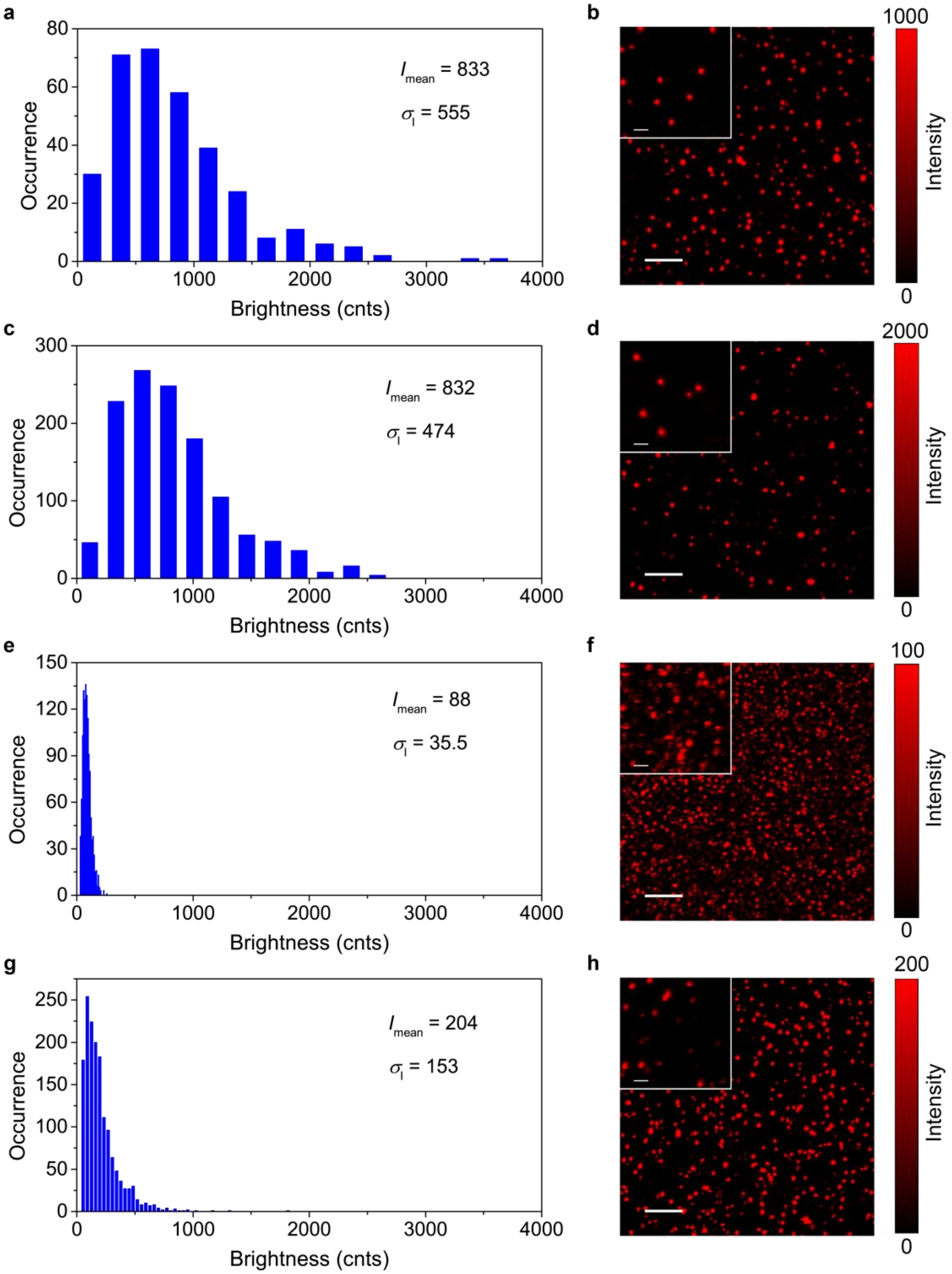


**Supplementary Figure 4. Fluorescence intensity obtained from individual Pdots and QDs.** Frequency histograms of integrated fluorescence intensities obtained from individual (a) PD1-L, (c) PD2-L, (e) QD605, and (g) QD655 deposited on coverslips. Fluorescence images obtained from individual (b) PD1-L, (d) PD2-L, (f) QD605, and (h) QD655 deposited on coverslips. All the images were recorded using a 532-nm excitation at the identical excitation power (1.5 kW $cm^{-2}$) and integration time (1 ms per pixel) and the same filter set. The insets show enlarged views. The mean fluorescence intensities and their standard deviations are summarized in Supplementary Table 2 and 3. Scale bars = 6 μm, Scale bars for insets = 1 μm.

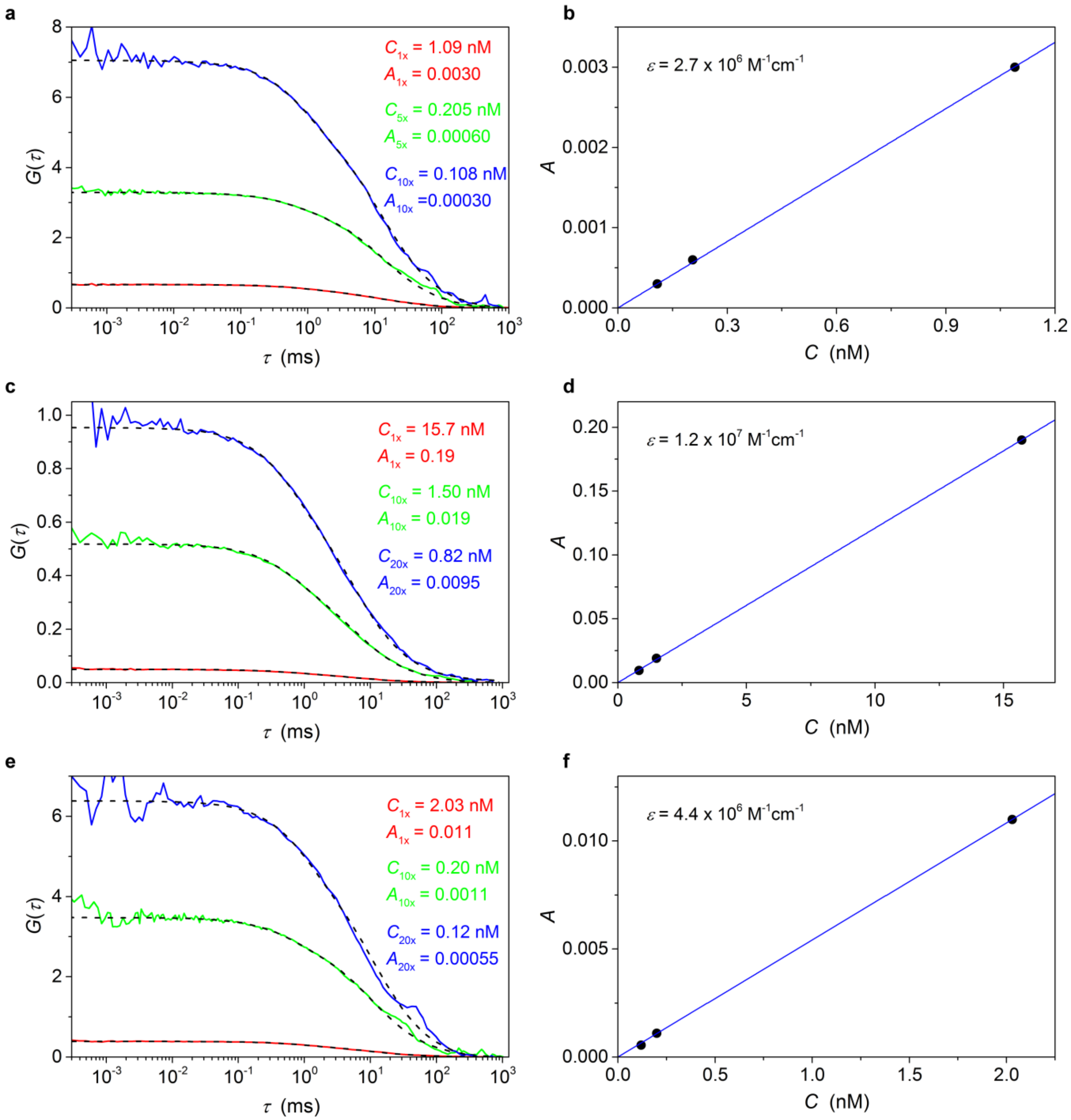


**Supplementary Figure 5. Determination of molar extinction coefficients ($\varepsilon$) of the Pdots using fluorescence correlation spectroscopy (FCS).** Autocorrelation curves obtained from (a) PD1-L, (c) PD2-L, and (e) PD2-H dispersed in water with three different dilutions. The concentrations of the Pdots (*C*) were calculated by fitting the autocorrelation curves to equation 1 (dashed lines). The $\varepsilon$ values were calculated from the peak absorptions (*A*) at each *C* determined by the FCS experiments. The autocorrelation curves were recorded using a 532-nm excitation at 1.5 kW cm$^{-2}$ power.

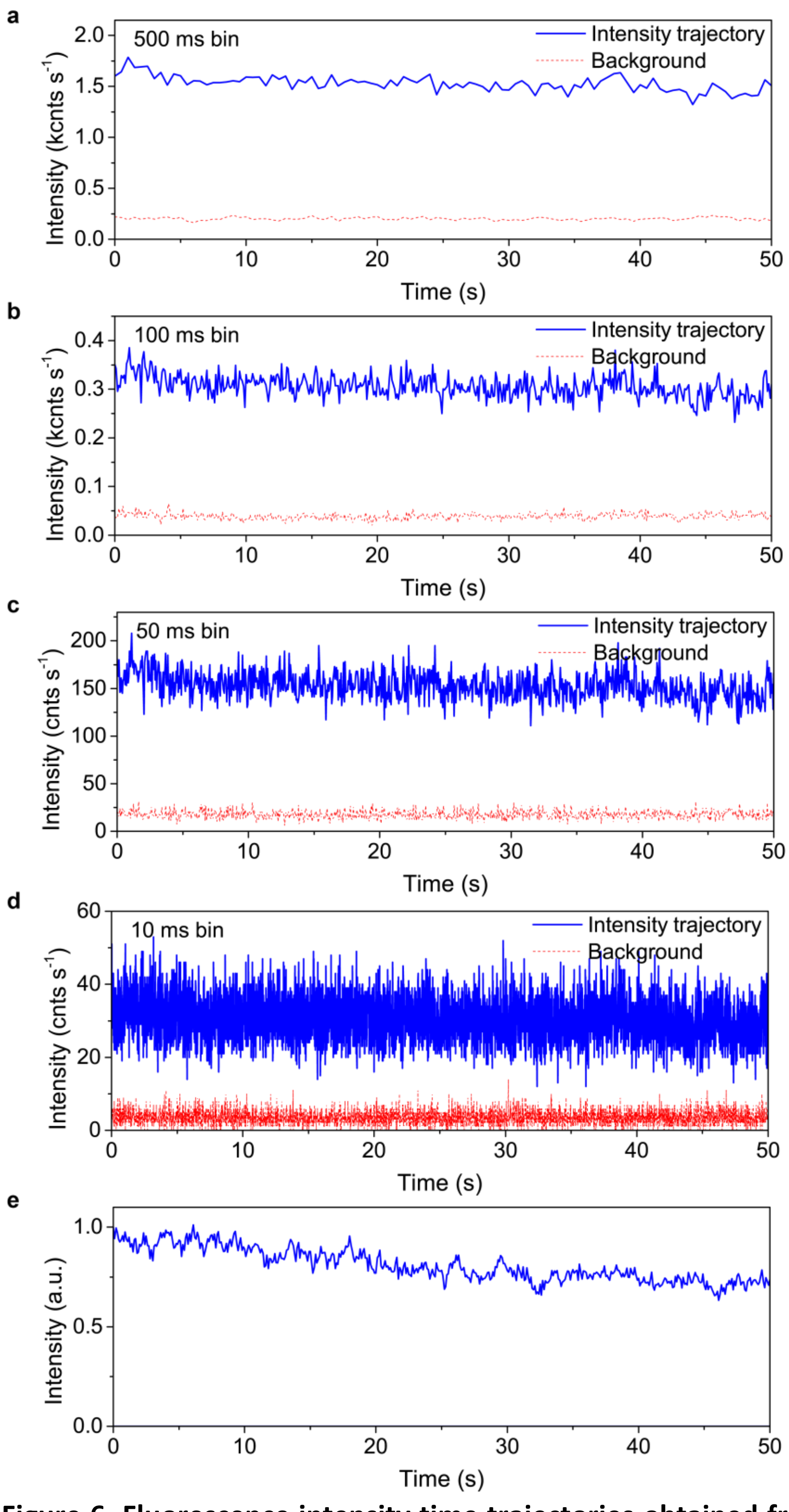


**Supplementary Figure 6. Fluorescence intensity time trajectories obtained from single Pdots.** (a-d) Intensity trajectories obtained from a single PD1-L particle drawn in bin sizes of (a) 500 ms, (b) 100 ms, (c) 50 ms, and (d) 10 ms. (e) Intensity trajectory obtained from a single PD2-L particle. The trajectories were measured using a 532-nm excitation at 1.5 kW $cm^{-2}$ power.

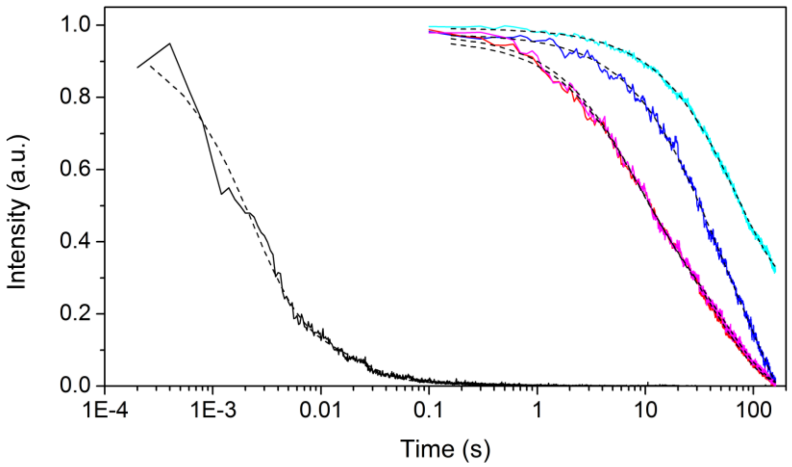


**Supplementary Figure 7. Photobleaching trajectories of the Pdots.** Fluorescence intensity trajectories of PD1-L (blue line), PD1-H (red line), PD2-L (cyan line), and PD2-H (magenta line) particles. All the trajectories were recorded using a 532-nm excitation at the excitation power of 15 kW $cm^{-2}$. The intensity trajectories were fitted to double-exponential decaying functions (dashed lines).

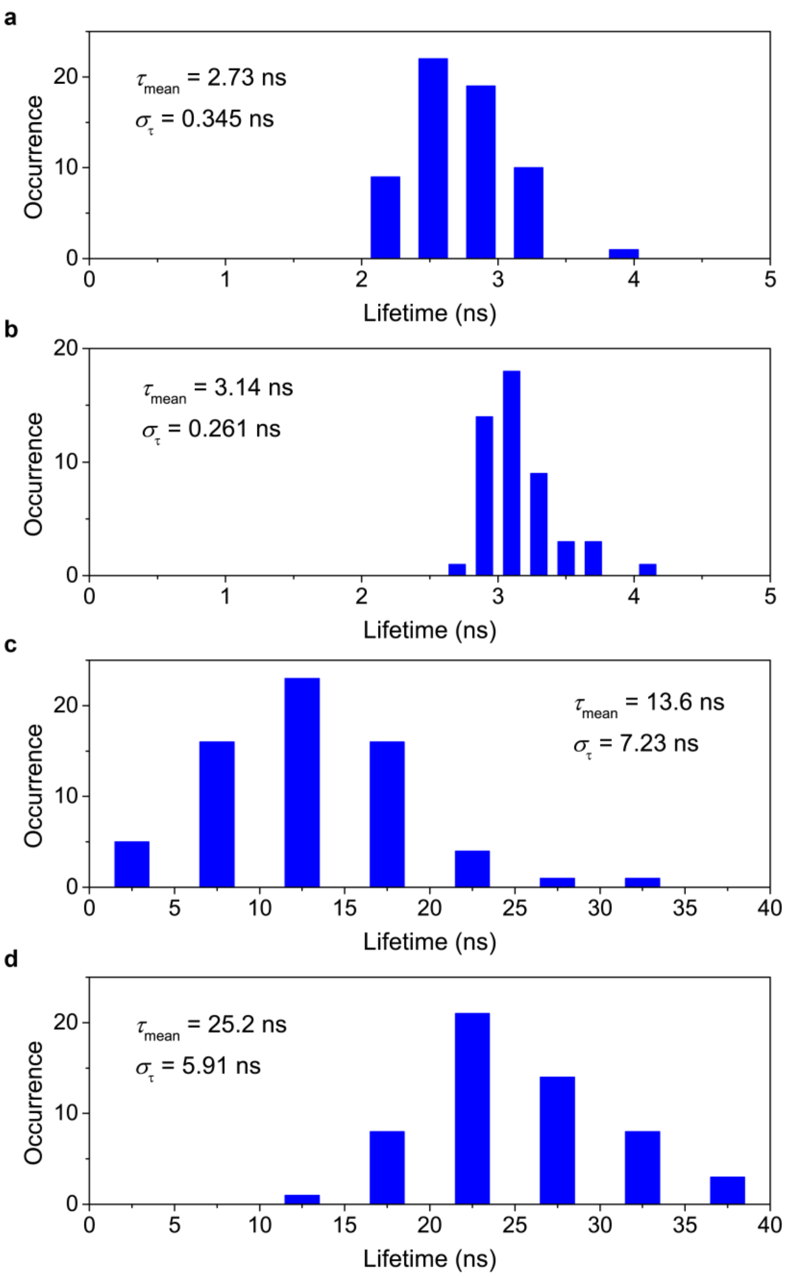


**Supplementary Figure 8. Frequency histograms of the mean photoluminescence lifetime of the Pdots and QDs.** Frequency histograms of the mean fluorescence lifetime obtained from the individual (a) PD1-L, (b) PD2-L, (c) QD605, and (d) QD655 particles. The fluorescence decay curves were fitted to double-exponential decaying functions. The mean lifetimes and their standard deviations are summarized in Table S3.

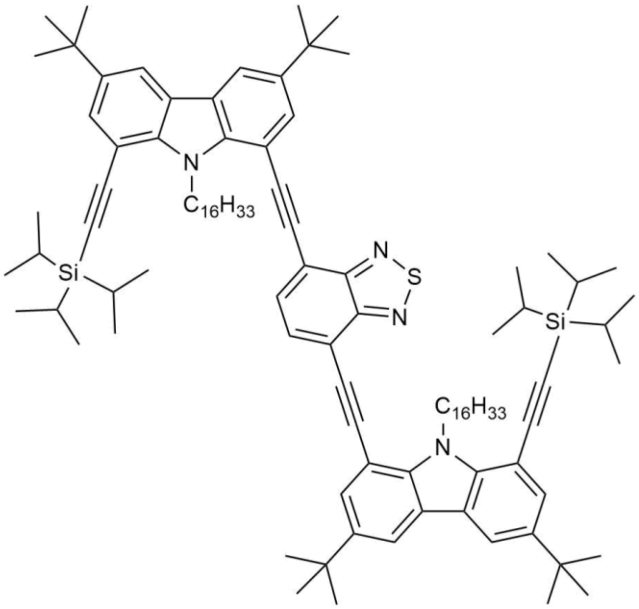


**Supplementary Figure 9. Chemical structure of the dimeric form of CzBT.**

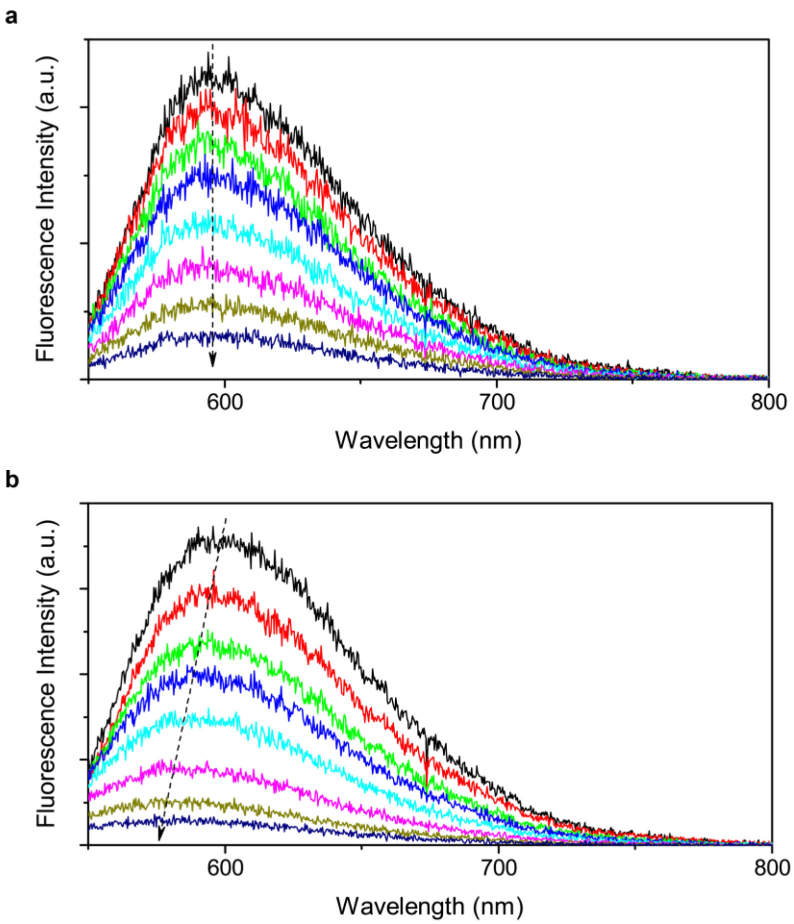


**Supplementary Figure 10. Time-lapse steady-state fluorescence spectra of the fabricated Pdots.** Fluorescence spectra of single (a) PD1-L and (b) PD1-H particles recorded during photobleaching. All the spectra were recorded using a 532-nm excitation at the excitation power of 15 kW cm$^{-2}$.

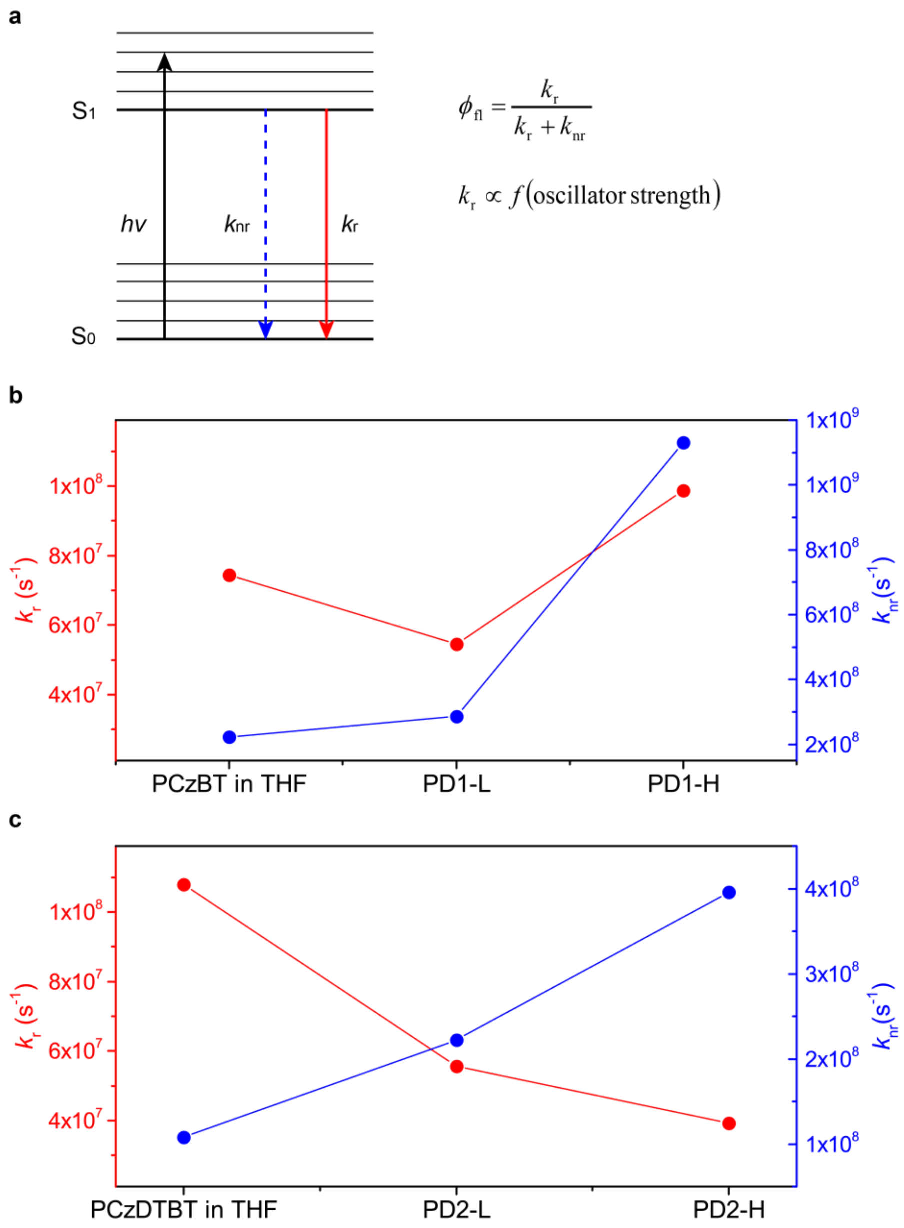


**Supplementary Figure 11. Kinetics of the excited-state deactivation of the fabricated Pdots.** (a) A Jablonski diagram describing radiative (red arrow) and non-radiative (blue arrow) deactivation paths of the excited state. (b) Radiative ($k_r$, red) and non-radiative ($k_{nr}$, blue) rate constants obtained for PCzBT in THF, PD1-L, and PD1-H. (c) Radiative ($k_r$, red) and non-radiative ($k_{nr}$, blue) rate constants obtained for PCzDTBT in THF, PD2-L, and PD2-H.

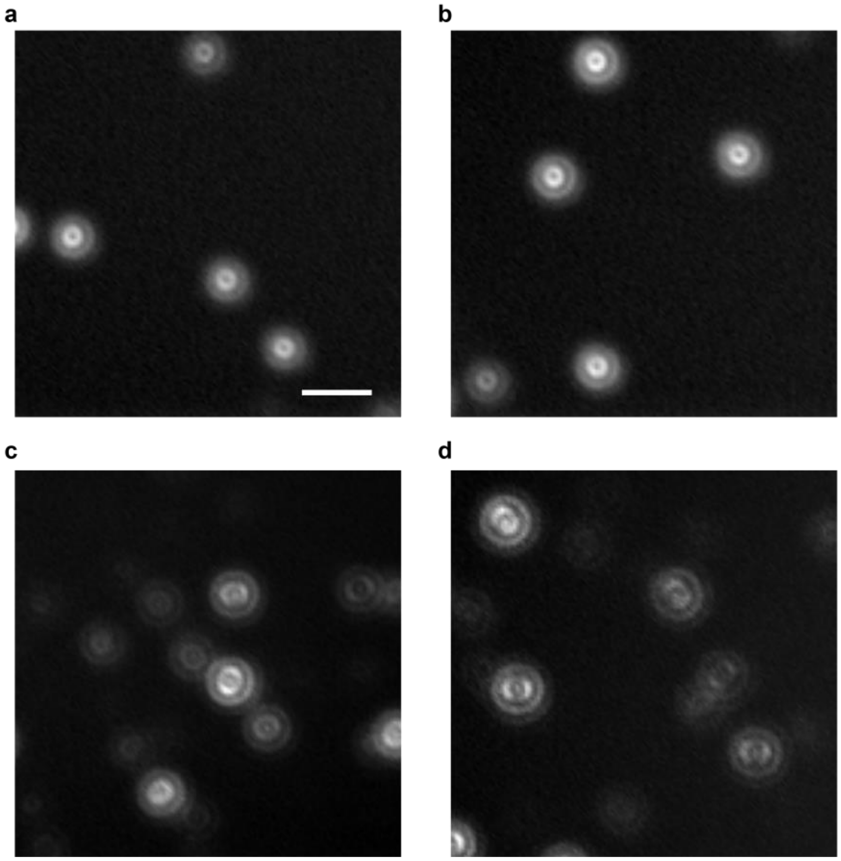


**Supplementary Figure 12. Defocused fluorescence images of the Pdots and QDs.** Defocused fluorescence images obtained from individual (a) PD1-L, (b) PD2-L, (c) QD605, and (d) QD655 particles deposited on a coverslip. Scale bar = 4 µm.

**Supplementary Table 1. Properties of fabricated Pdots.**

| Sample | $\lambda_{ab}$ (nm) | $\lambda_{fl}$ (nm) | $\varepsilon$ ($M^{-1}cm^{-1}$) | $\phi_{fl}$ | $\tau_{fl}$ (ns) | $\phi_{bl}$ | Size (nm)[a] | ζ (mV)[b] |
|---|---|---|---|---|---|---|---|---|
| PCzBT[c] | 482 | 608 | $1.15 \times 10^5$ | 0.25 | 3.4 | N/A | N/A | N/A |
| PD1-L[d] | 497 | 631 | $(2.8 \pm 0.55) \times 10^6$ | 0.16 | 2.9 | $(3.3 \pm 0.31) \times 10^{-11}$ | $3.0 \pm 0.56$ | -54 |
| PD1-H[d] | 490 | 600 | N/A | 0.08 | 0.81 | $(5.3 \pm 0.49) \times 10^{-11}$ | $3.1 \pm 0.56$ | -55 |
| PCzDTBT[c] | 494 | 652 | $2.7 \times 10^4$ | 0.50 | 4.6 | N/A | N/A | N/A |
| PD2-L[d] | 503 | 660 | $(1.2 \pm 0.12) \times 10^7$ | 0.20 | 3.6 | $(1.7 \pm 0.16) \times 10^{-11}$ | $4.5 \pm 0.97$ | -51 |
| PD2-H[d] | 493 | 674 | $(4.4 \pm 0.45) \times 10^6$ | 0.09 | 2.3 | $(1.2 \pm 0.11) \times 10^{-10}$ | $5.7 \pm 1.3$ | -52 |
| QD605 | N/A | 605 | $5.8 \times 10^5$ [e] | 0.2[f] | 13.7 | N/A | $6.4 \pm 0.60$ | N/A |
| QD655 | N/A | 655 | $2.4 \times 10^6$ [e] | 0.15[g] | 20.1 | N/A | $(6.7 \pm 0.66) \times (13 \pm 1.49)$ | N/A |

*a*: diameter of the particles determined by TEM, *b*: zeta potential of the colloidal particles dispersed in water, *c*: measured in THF, *d*: measured in water, *e*: molar extinction coefficients at 532 nm, *f*: literature value reported in Supplementary Reference 1,[1] *g*: literature value reported in Supplementary Reference 2.[2]

**Supplementary Table 2. Fluorescence brightness and size of the fabricated Pdots.**

| Sample | Brightness | Size (nm) | Volume ($nm^3$) | Brightness per unit volume |
|---|---|---|---|---|
| PD1-L | 833 | 3.0 | 14 | 60 |
| PD2-L | 832 | 4.5 | 48 | 17 |
| QD605 | 88 | 6.4 | 137 | 0.64 |
| QD655 | 204 | 6.7 × 13 | 306 | 0.67 |

**Supplementary Table 3. Distribution of fluorescence brightness and lifetime determined by the single-particle fluorescence microscopy experiments.**

| Sample | $I_{mean}$ [a] | $\sigma_I$ [b] | $\sigma_I/I_{mean}$ [c] | $\tau_{mean}$ (ns) [d] | $\sigma_\tau$ (ns) [e] | $\sigma_\tau/\tau_{mean}$ [f] |
|---|---|---|---|---|---|---|
| PD1-L | 833 | 555 | 0.666 | 2.73 | 0.345 | 0.126 |
| PD2-L | 832 | 474 | 0.570 | 3.14 | 0.261 | 0.0831 |
| QD605 | 88 | 35.5 | 0.404 | 13.6 | 7.23 | 0.530 |
| QD655 | 204 | 153 | 0.749 | 25.2 | 5.91 | 0.234 |

*a*: mean fluorescence intensity, *b*: standard deviation of the intensity, *c*: normalized standard deviation of the intensity, *d*: mean fluorescence lifetime, *e*: standard deviation of the lifetime, f: normalized standard deviation of the lifetime.

**Supplementary References:**


1. Wu, Y., Lopez, G. P., Sklar, L. A. & Buranda, T. Spectroscopic characterization of streptavidin functionalized quantum dots. *Anal Biochem* **364**, 193-203 (2007).

2. Ding, D. *et al.* Bright Far-Red/Near-Infrared Conjugated Polymer Nanoparticles for In Vivo Bioimaging. *Small* **9**, 3093-3102 (2013).
